# Allosterism vs. Orthosterism: Recent Findings and Future Perspectives on A_2B_ AR Physio-Pathological Implications

**DOI:** 10.3389/fphar.2021.652121

**Published:** 2021-03-24

**Authors:** Elisabetta Barresi, Claudia Martini, Federico Da Settimo, Giovanni Greco, Sabrina Taliani, Chiara Giacomelli, Maria Letizia Trincavelli

**Affiliations:** ^1^ Department of Pharmacy, University of Pisa, Pisa, Italy; ^2^ Department of Pharmacy, University of Naples “Federico II”, Naples, Italy

**Keywords:** adenosine receptors, allosteric modulators, A_2B_ receptor, mesenchymal stromal cells, bone healing

## Abstract

The development of GPCR (G-coupled protein receptor) allosteric modulators has attracted increasing interest in the last decades. The use of allosteric modulators in therapy offers several advantages with respect to orthosteric ones, as they can fine-tune the tissue responses to the endogenous agonist. Since the discovery of the first A_1_ adenosine receptor (AR) allosteric modulator in 1990, several efforts have been made to develop more potent molecules as well as allosteric modulators for all adenosine receptor subtypes. There are four subtypes of AR: A_1_, A_2A_, A_2B_, and A_3_. Positive allosteric modulators of the A_1_ AR have been proposed for the cure of pain. A_3_ positive allosteric modulators are thought to be beneficial during inflammatory processes. More recently, A_2A_ and A_2B_ AR allosteric modulators have also been disclosed. The A_2B_ AR displays the lowest affinity for its endogenous ligand adenosine and is mainly activated as a consequence of tissue damage. The A_2B_ AR activation has been found to play a crucial role in chronic obstructive pulmonary disease, in the protection of the heart from ischemic injury, and in the process of bone formation. In this context, allosteric modulators of the A_2B_ AR may represent pharmacological tools useful to develop new therapeutic agents. Herein, we provide an up-to-date highlight of the recent findings and future perspectives in the field of orthosteric and allosteric A_2B_ AR ligands. Furthermore, we compare the use of orthosteric ligands with positive and negative allosteric modulators for the management of different pathological conditions.

## Introduction

G-protein-coupled receptors (GPCRs) are a large family of membrane receptors that mediate the response to several extracellular stimuli. Thus, several efforts have been made to discover molecules acting on GPCRs that represent about one-fourth of marketed drugs approved in 2019 by the FDA (Salmaso and Jacobson, 2020). Among the GPCR family, Adenosine receptors (ARs) are mainly involved in the sensing of tissue damage rather than homeostatic regulators under physiological conditions (Xiao et al., 2019). Four subtypes of ARs (A_1_, A_2A_, A_2B_, and A_3_) mediate the response to the increase of extracellular adenosine concentrations in response to stressors (Chen et al., 2013). Adenosinergic pathways possess a dual face: on one side, elevated adenosine concentration restores an energy imbalance; on the other side, chronic exposure to high adenosine levels can switch to the promotion of pathological conditions such as uncontrolled inflammation, cytokine release, and fibrosis (Borea et al., 2017).

Particularly, the A_2B_ AR has emerged as a possible therapeutic target in several physio-pathological conditions, including asthma (Cicala and Ialenti, 2013), colitis (Kolachala et al., 2008), cancer (Gao and Jacobson, 2019), cardiovascular, and metabolic disorders (Effendi et al., 2020). However, among all the ARs, the A_2B_ subtype is the least characterized from a pharmacological point of view owing to the lack of X-ray structure and to its low affinity for the prototypic standard ligands commonly used to study ARs. Of note, the four AR subtypes, the A_1_, A_2A_, A_2B_, and A_3_, share a highly conserved binding (the “orthosteric” one) site of the endogenous agonist adenosine challenging the design of selective agonists. The design and development of positive (PAMs) as well as negative (NAMs) allosteric modulators binding to a less conserved, and topographically distinct site have emerged as an attractive strategy. Allosteric modulators can modulate the effects of the endogenous ligand in its site of production evidencing their ability to be spatially and temporally more selective than the orthosteric ones (Changeux and Christopoulos, 2016; Gregory et al., 2019; Congreve et al., 2020).

### Biological Effects of the A_2B_ AR

**FIGURE 1:**
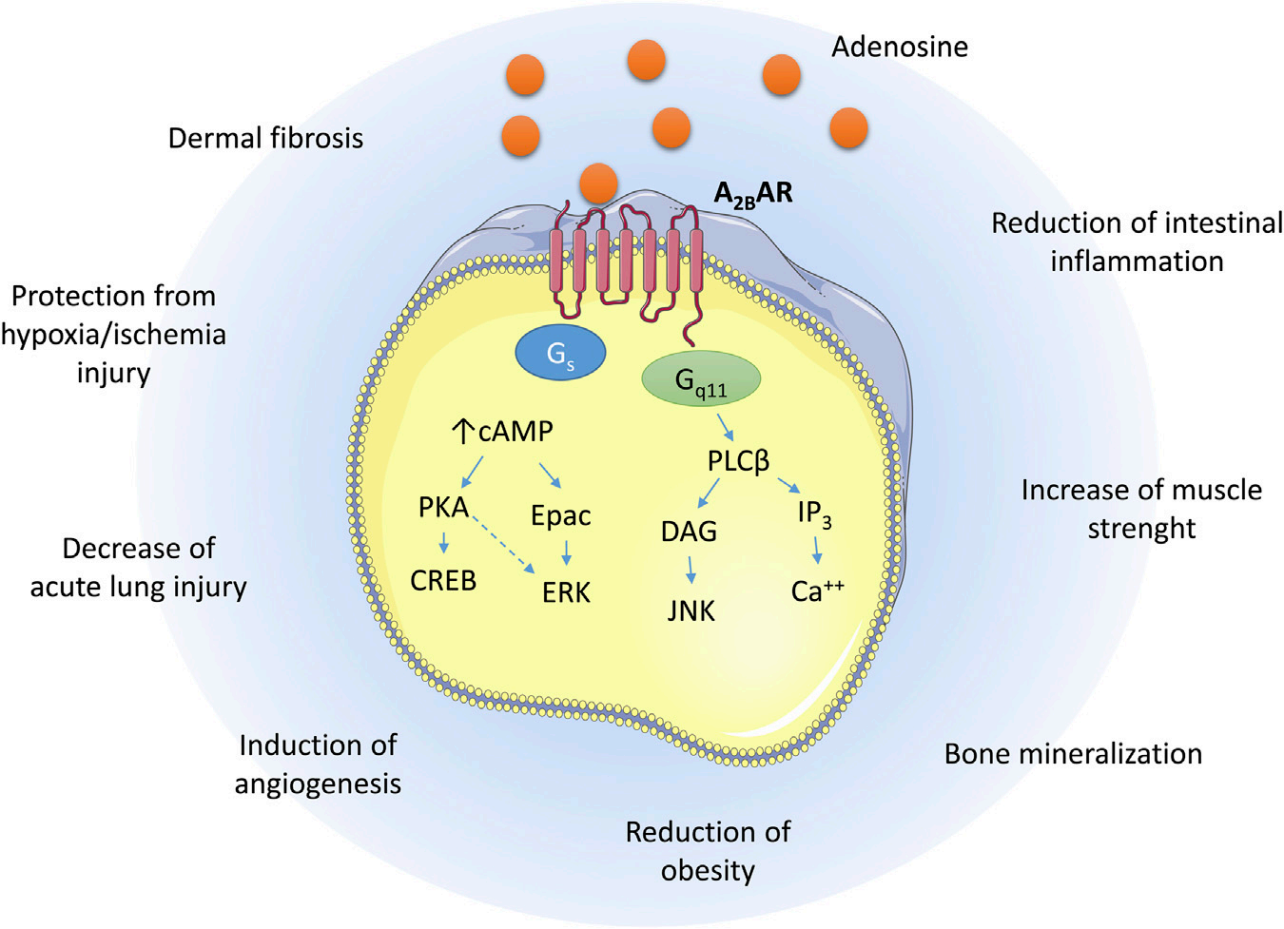
Schematic representation of the A_2B_ AR signaling pathways upon adenosine binding and the biological effects exerted in different tissues.

The coupling of the A_2B_ AR with Gs causes, within the cells, the activation of adenylate cyclase with a consequent increase of cAMP levels. The increase of cAMP concentrations activates the protein kinase A (PKA) and Epac leading to the phosphorylation of cAMP response element-binding protein (CREB) and extracellular signal-regulated kinases (ERK) (Schulte and Fredholm, 2003; Giacomelli et al., 2018). Furthermore, the A_2B_ AR is coupled with G_q11_ that mediates the activation of Phospholipase C (PLC) to increase the levels of 1,4,5-inositol triphosphate (IP_3_)/diacylglycerol (DAG), leading to activation of Protein kinase C (PKC) and increase of Ca^2+^ levels (Figure 1) (Ciruela et al., 2010).

Although there are no A_2B_ AR ligands currently in clinical evaluation, several pre-clinical data have been reported to support their use to treat different conditions such as acute lung injury, ischemia, vascular leakage, metabolic disorders, and bone defects (Jacobson et al., 2019; Carluccio et al., 2020; Gnad et al., 2020) (Table 1). The A_2B_ AR is highly expressed in the respiratory tract and its modulation has been related to the pathogenesis of chronic obstructive pulmonary disease (COPD) and pulmonary fibrosis (Zhong et al., 2005; Cronstein, 2011; Giacomelli et al., 2018). The A_2B_ AR has been proposed as a potential target in acute lung injury (ALI): in fact, the administration of aerosolized BAY-60-6583 **1** attenuates pulmonary edema and diminishes lung inflammation (Hoegl et al., 2015). The A_2B_ AR expression has been related to the activation of the hypoxia-inducible factor in different cell types such as endothelial cells (Feoktistov et al., 2004), lung (Eckle et al., 2014), liver cancer cells (Kwon et al., 2019), intestinal epithelial cells (Kong et al., 2006). Its activation has been reported to be useful in the treatment of ischemic injury in different tissues such as the intestines (Hart et al., 2011), heart (Ni et al., 2018) and brain (Coppi et al., 2020).

**TABLE 1:** Representative orthosteric ligands in pre-clinical and clinical trials and allosteric modulators of A_2B_ AR.

Compound	Structure	Class	Selectivity	Activity
BAY-60-6583 **1**	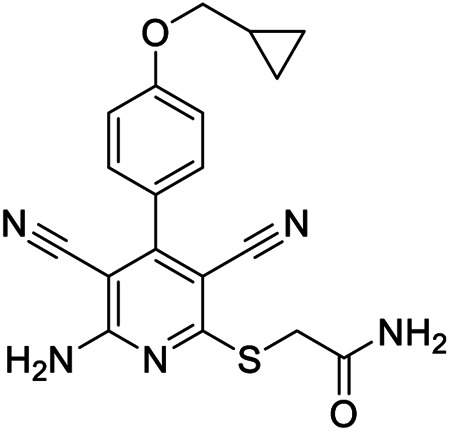	Orthosteric agonist	A_2B_	EC_50_ (hA_2B_) = 3 nM
CVT-6883 **2**	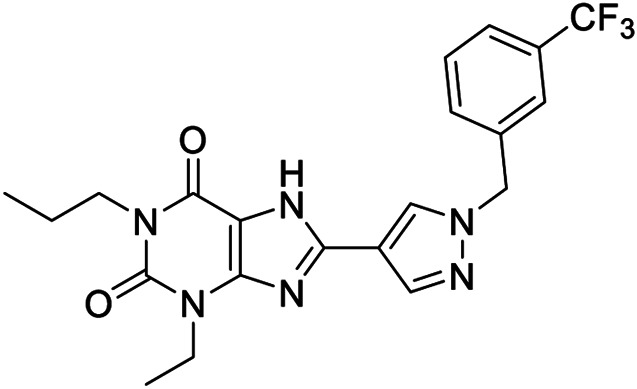	Orthosteric antagonist	A_2B_	*K*_*i*_ (hA_2B_) = 8.3 nM
CGS-15493 **3**	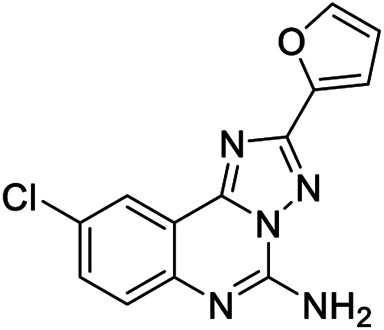	Orthosteric antagonist	Not selective	*K*_*i*_ (rA_1_) = 21 nM (rA_2A_) = 3.3 nM (rA_2B_) = 16.4 nM (rA_3_) = 190 nM
IPDX **4**	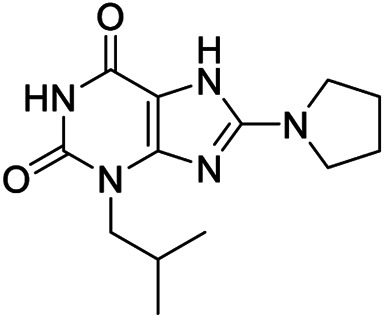	Orthosteric antagonist	A_2B_	*K*_*i*_ (hA_2B_) = 625 nM
QAF-807 **5**	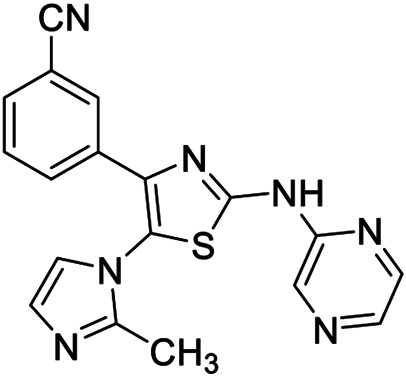	Orthosteric antagonist	Not selective	*K*_*i*_ (hA_1_) = 197 nM (hA_2A_) = 1.670 nM (hA_2B_) = 3 nM (hA_3_) = 10 nM
**6a,b**	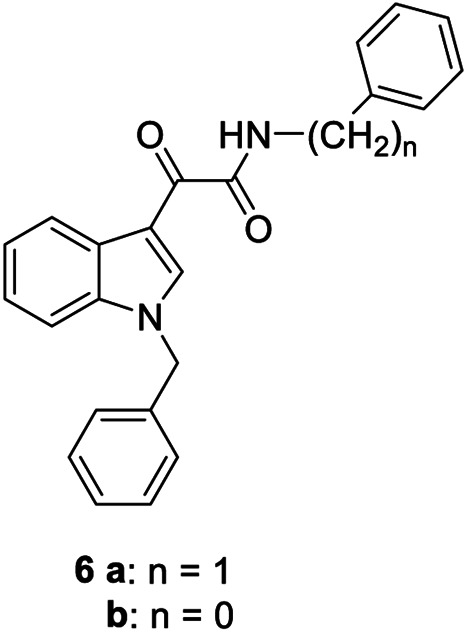	Positive allosteric modulators	A_2B_	**6a** EC_50_ (hA_2B_)^a^ = 427 nM **6b** EC_50_ (hA_2B_)^a^ = 445 nM
**7a**	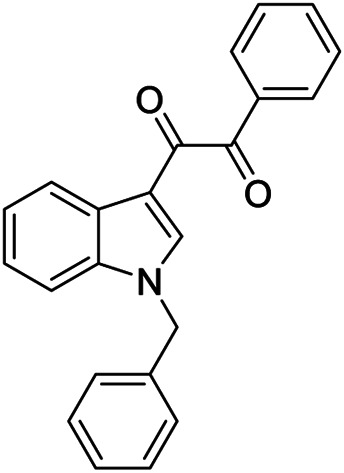	Positive allosteric modulator	A_2B_	EC_50_ (hA_2B_)^a^ = 249 nM
**7b,c**	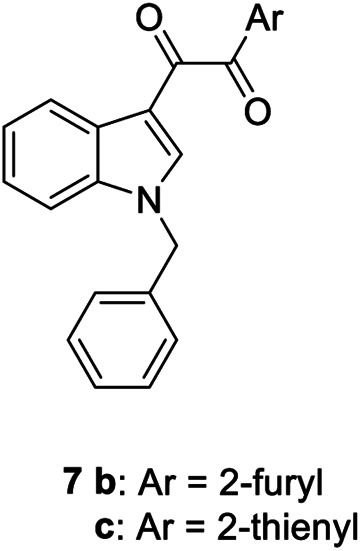	Negative allosteric modulators	A_2B_	**7b** IC_50H_ (hA_2B_)^b^ = 0.4 nM IC_50L_ (hA_2B_)^b^ = 1.550 nM **7c** IC_50_ (hA_2B_)^b^ = 2.5 nM
**8a,b**	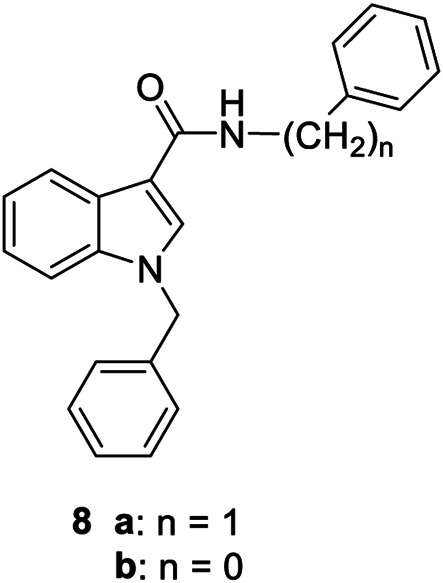	Negative allosteric modulators	A_2B_	**8a** IC_50H_ (hA_2B_)^b^ = 0.2 nM IC_50L_ (hA_2B_)^b^ = 1.050 nM **8b** IC_50H_ (hA_2B_)^b^ = 0.4 nM IC_50L_ (hA_2B_)^b^ = 420 nM
**9**	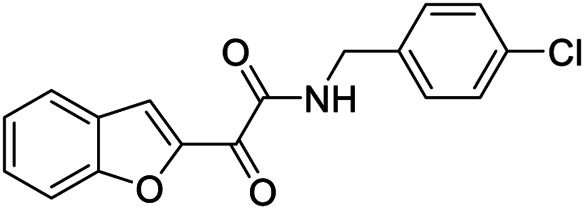	Positive allosteric modulator	A_2B_	EC_50_ (hA_2B_)^a^ = 636 nM

^a^ CHO cells stably transfected with hA_2B_ AR were treated with a fixed EC_50_ NECA concentration (100 nM) in the absence or presence of different concentrations of the tested compound. The EC_50_ values to promote the cAMP accumulation were reported.

^b^ CHO cells stably transfected with hA_2B_ AR were treated with a fixed EC_50_ NECA concentration (100 nM) in the absence or presence of different concentrations of the tested compound. The IC_50_ values to inhibit the cAMP accumulation were reported.

In addition to the already known A_2B_ AR activity, the A_2B_ AR has been recently related to physiological processes and pathological conditions opening the way to new possible therapeuthic applications of the A_2B_ AR ligands. In fact, the activity of the A_2B_ AR has been linked to glucose homeostasis and insulin secretion and resistance (Merighi et al., 2015). Accordingly, it has been reported that the A_2B_ AR is abundantly expressed in skeletal muscle (SKM) as well as brown adipose tissue (BAT), and its gene deletion in SKM cells causes sarcopenia, diminishes muscle strength, and reduces energy expenditure (Gnad et al., 2020). Similarly, the adipose tissue specific silencing exacerbates the age-related reduction of BAT. The same authors demonstrated that the receptor stimulation with the A_2B_ AR agonist BAY-60-6583 **1** ameliorates obesity (Gnad et al., 2020).

Elevated adenosine concentration is known to exert immunosuppressive action through activation of the ARs mainly by the A_2A_ and A_2B_ subtypes. Interestingly, the use of adenosine as A_2A_ and A_2B_ AR agonist has been proposed and tested as an effective treatment strategy for the recent COVID-19 (Correale et al., 2020; Falcone et al., 2020).

The role of the different ARs in orchestrating the mesenchymal stem cell (MSCs) differentiation has been the focus of several researches (Reviewed in Carluccio et al., 2020). The activation of the A_1_ AR promotes osteoclast differentiation reducing the MSC-osteoblast differentiation. Conversely, several evidence support the role played by the A_2B_ AR in the adenosine-mediated commitment of MSC into osteoblast differentiation (Gharibi et al., 2011; Carroll et al., 2012; He et al., 2013; Mediero and Cronstein, 2013; Rao et al., 2015; Shih et al., 2019). The receptor activation promotes the expression of the osteogenic factor Runx2 and the phosphatase alkaline (ALP), favoring osteoblastogenesis (Gharibi et al., 2011). Adenosine levels raise up to micromolar concentrations during bone injury, thus triggering the activation of the A_2B_ AR that is highly expressed in MSCs. Furthermore, the inflammation evoked during bone injury further enhances the anabolic responses evoked by A_2B_ AR ligands (Daniele et al., 2017). These data support the role of the A_2B_ AR as an interesting target in the treatment of bone defects such as osteoporosis. Accordingly, the A_2B_ AR stimulation attenuates bone loss in ovariectomized mice, supporting its potential as a target for osteoporosis also consequent to estrogen deficiency (Shih et al., 2019).

### Adenosine A_2B_ AR Orthosteric vs Allosteric Ligands

It is beyond the scope of this perspective to review the whole available literature concerning A_2B_ AR ligands. Instead, we have chosen to select a number of relevant (classes of) compounds to 1) offer a reliable overview of those drugs involved in both preclinical and clinical studies, and 2) briefly recapitulate the effects produced by a specific orthosteric or allosteric binding to the A_2B_ AR.

The number of A_2B_ AR agonists developed is the most limited among the ARs agonists, and no agonists for this AR subtype have yet entered clinical trials; nevertheless, some relevant A_2B_ AR agonists have been proposed and subjected to preclinical studies. Among them, the most promising compound, the non-nucleoside agonist BAY-60-6583 **1** (Table 1), showed to be useful in the treatment of acute lung injury as well as in cardiovascular diseases, such as atherosclerosis and coronary artery disorders (Eckle et al., 2008; Gao et al., 2014).

Conversely, several highly potent and selective A_2B_ AR antagonists have been reported and some of these had entered in clinical trials in the past decades. CVT-6883 (**2**, Table 1, also known as GS-601), developed by Gilead along with various other compounds, such as, CGS15493 (**3**, Table 1, developed by Novartis), WO-00125210 (Bayer HealthCare Pharmaceuticals), IPDX (**4**, Table 1, Vanderbilt University), and ATL-907 (Adenosine Therapeutics) have entered phase I and II trials for the treatment of asthma (Chandrasekaran et al., 2019; Effendi et al., 2020). In addition, the dual A_2B_/A_3_ AR antagonist QAF-807 (**5**, Table 1, Novartis) had reached phase III clinical trial but it failed to attenuate PC20 AMP challenge as a marker of airway inflammation in mild asthmatic subjects (Wilson, 2008).

The discrepancy between the few drugs entered in clinical practice and the pivotal role of adenosinergic system in several pathological processes can be partially explained by the ubiquitous AR expression in almost all tissues, increasing the possibility of unwanted side effects. In this respect, small-molecules acting as allosteric modulators of the A_2B_ AR might be worthy of investigation as they could represent a potential novel therapeutic strategy for pathological conditions characterized by an altered functionality of this AR subtype.

The first selective PAM, PD81,723, has been reported in 1990 for the A_1_ AR subtype (Bruns and Fergus, 1990). Since then, many research groups have performed extensive structure-activity relationship studies and reported several PAMs of different AR subtypes (Valant et al., 2012; Jacobson et al., 2018; Deb et al., 2019). Less has been reported regarding A_2A_ and A_2B_ AR allosteric modulators. In 2013, our research group serendipitously has discovered the first class of A_2B_ AR allosteric modulators (Taliani et al., 2013). Specifically, adopting the strategy of designing AR antagonists starting from benzodiazepine receptor (BzR) ligands, the indol-3-ylglyoxylamide scaffold, previously exploited by us to develop BzR ligands (Da Settimo et al., 1996; Da Settimo et al., 1998; Primofiore et al., 2001; Primofiore et al., 2006; Primofiore et al., 2007; Taliani et al., 2009; Cosimelli et al., 2012; Salerno et al., 2012), has been structurally modified, providing a series of 1-benzyl-3-ketoindoles (**6a**-**b**, **7a**-**c**, **8a**-**b**, Table 1). These compounds possess two structural features characterizing most of the A_2B_ AR antagonists: three lipophilic moieties linked to a heterocyclic ring and a group capable to establish hydrogen bonds.

No compounds show significant binding affinities toward A_1_, A_2A,_, and A_3_ ARs, except for **7a** and **7b**, which display moderate A_1_ AR affinity (sub-micromolar K_i_ values). Quite surprisingly, the new compounds act as selective human A_2B_ AR modulators in a stably transfected cell line. In particular, **6a**,**b** and **7a** behave as PAMs of the A_2B_ AR in functional assay by increasing the efficacy but not the potency of the A_2B_ AR agonists (NECA, BAY 60-6583, adenosine) in stimulating cAMP accumulation. These compounds have been deeply investigated using competitive and kinetic binding experiments resulting in the hypothesis that they favor the receptor active state without altering the orthosteric site. Compounds **7b**,**c** and **8a**,**b** act as NAMs of the A_2B_ AR by decreasing both efficacy and potency of agonists. Similar to the PAMs, the activity of these compounds have been also investigated with binding experiments. They probably interfer with receptor-G_s_ protein coupling and, consequently, with the agonist functional response favoring the receptor uncoupled state (Trincavelli et al., 2014).

The positive or negative profile of these compounds seems to be correlated to small structural differences. However, the limited number of compounds allowed to delineate only preliminary structure-activity relationships. In brief, compounds **6a**, **6b**, **8a**, and **8b** act as PAMs or NAMs depending on the nature of the linker between the indole nucleus and the lipophilic side chain, i.e. glyoxylamide for **6** and carboxamide for **8**. Conversely, for compounds **7** the interaction with the protein strictly depends on the nature of the pendant aromatic ring: **7a** with a phenyl ring is a PAM while **7b** and **7c**, featuring a furyl or a thienyl ring, respectively, are NAMs.

Based on the evidence that the A_2B_ AR is the principal AR subtype implicated in MSC differentiation to osteoblasts and bone formation (Gharibi et al., 2011), the A_2B_ AR PAM **6b** has been selected for its specificity toward the A_2B_ AR and evaluated for its effect on the agonist-mediated MSC differentiation to osteoblasts. Compound **6b** potentiates the effects of either adenosine and synthetic orthosteric A_2B_ AR agonists (NECA and BAY 60-6583 **1**) in promoting MSC differentiation to osteoblasts *in vitro* (Trincavelli et al., 2014b) by increasing the osteogenic marker expression and by favoring osteoblast mineralization. In addition, in the early stage of differentiation, **6b** potentiates the physiological and A_2B_ agonist-mediated reduction of IL-6 levels that in turn is necessary to support the differentiation process in MSCs. On the contrary, in the late differentiation phase, **6b** improves the physiological and A_2B_ agonist-mediated IL-6 increase, important to ensure a pro-survival effect in osteoblasts. These results provide the basis for a prospective therapeutic use of selective A_2B_ AR PAMs in bone diseases.

Very recently, Barresi et al. (2021), to expand the knowledge about the pharmacophoric requirements for A_2B_ AR allosteric modulation, have reported a series of novel derivatives chemically related to **6–8**. Compounds from this library exhibit different degrees of similarity with the indoles **6**, **7**, and **8**, including novel indole-based derivatives bearing various substitutions at 1- and/or 3-positions, and derivatives characterized by different aromatic heterocycles in place of indole. Interestingly, structure-activity relationships in terms of matrix mineralization stimulation activity in MSCs (either in the presence or in the absence of the agonist BAY60-6583 **1**) suggest that the indole nucleus and N1-arylalkyl group do not represent key pharmacophoric elements for a compound acting as A_2B_ AR PAM. In this study compound **9** (Table 1), which possesses a peculiar chemical structure with respect to the reference *N*1-benzyl substituted indoles **6–8**, has been identified to represent a novel lead structure for the development of novel A_2B_ AR PAMs potentially acting as anti-osteoporosis agents.

## Discussion

Allosteric modulation is a fundamental mechanism in biology and the development of allosteric ligand on GPCRs is a fast-growing field. Allosteric modulators have the potential to inhibit, activate, or maintain the signaling of the GPCR receptor allowing their modulation based on the physiological requirement without blocking endogenous ligand binding. The research on this field has delivered hundred of candidates in the pipeline and also FDA-approved therapies.

Among the GPCRs, ARs have attracted considerable attention in drug developmemt. During the last fifty decades, several efforts have been made to develop small molecules acting as agonists, antagonists or allosteric enhancers of ARs. Most of these molecules failed in clinical trials and only three are currently approved for human use: adenosine, Regadenoson and Istradefylline. However, increasing evidence demonstrates the biological role played by ARs, particularly by the A_2B_ subtype, in pathological conditions. The A_2B_ AR is expressed under stress conditions in almost all human tissues, and exhibits a low affinity for its endogenous agonist adenosine. Based on the tissue, the nature of the stimuli and the time of exposure, the A_2B_ AR can mediate positive or negative effects. Thus, in the last decade, the research has been focused, on one hand, on a deeper knowledge of the molecular mechanism evoked by the A_2B_ AR activation in different pathological conditions; on the other hand, on the development of small molecules able to selectively bind to this receptor subtype. New A_2B_ AR ligands are currently being developed and the discovery of allosteric modulators represents an attractive research topic. The use of a GPCR orthosteric agonist can always lead to undesired effects due to its activation in other tissues. The limitation of the use of an AR orthosteric agonists and antagonists can not be completely overcome by the use of an allosteric modulator. Of note, the use of an A_2B_ AR agonist can lead to its activation in all the tissue (also where a low amount of adenosine is present) promoting undesired side effects. Conversely, allosteric modulators may represent a more physiologic alternative to orthosteric agonist and antagonist as they promote site-specific and event-specific responses mainly in damaged tissues, where adenosine is massively released. Thus, the development of potent and selective A_2B_ AR PAMs can open the way to a new application of AR ligand in clinical practice likely reducing the side effects with respect to those potentially caused by orthosteric agonists.

## Data Availability

The original contributions presented in the study are included in the article/Supplementary Material, further inquiries can be directed to the corresponding authors.
